# Leukocyte nucleus segmentation and nucleus lobe counting

**DOI:** 10.1186/1471-2105-11-558

**Published:** 2010-11-12

**Authors:** Yung-Kuan Chan, Meng-Hsiun Tsai, Der-Chen Huang, Zong-Han Zheng, Kun-Ding Hung

**Affiliations:** 1 Department of Management Information Systems, National Chung Hsing University, No. 250, Kuokuang Rd., Taichung, R.O.C, Taiwan; 2 Department of Computer Science and Engineering, National Chung Hsing University, No. 250, Kuokuang Rd., Taichung, R.O.C, Taiwan

## Abstract

**Background:**

Leukocytes play an important role in the human immune system. The family of leukocytes is comprised of lymphocytes, monocytes, eosinophils, basophils, and neutrophils. Any infection or acute stress may increase or decrease the number of leukocytes. An increased percentage of neutrophils may be caused by an acute infection, while an increased percentage of lymphocytes can be caused by a chronic bacterial infection. It is important to realize an abnormal variation in the leukocytes. The five types of leukocytes can be distinguished by their cytoplasmic granules, staining properties of the granules, size of cell, the proportion of the nuclear to the cytoplasmic material, and the type of nucleolar lobes. The number of lobes increased when leukemia, chronic nephritis, liver disease, cancer, sepsis, and vitamin B12 or folate deficiency occurred. Clinical neutrophil hypersegmentation has been widely used as an indicator of B12 or folate deficiency.Biomedical technologists can currently recognize abnormal leukocytes using human eyes. However, the quality and efficiency of diagnosis may be compromised due to the limitations of the biomedical technologists' eyesight, strength, and medical knowledge. Therefore, the development of an automatic leukocyte recognition system is feasible and necessary. It is essential to extract the leukocyte region from a blood smear image in order to develop an automatic leukocyte recognition system. The number of lobes increased when leukemia, chronic nephritis, liver disease, cancer, sepsis, and vitamin B12 or folate deficiency occurred. Clinical neutrophil hypersegmentation has been widely used as an indicator of B12 or folate deficiency.

**Results:**

The purpose of this paper is to contribute an automatic leukocyte nuclei image segmentation method for such recognition technology. The other goal of this paper is to develop the method of counting the number of lobes in a cell nucleus. The experimental results demonstrated impressive segmentation accuracy.

**Conclusions:**

Insensitive to the variance of images, the LNS (Leukocyte Nuclei Segmentation) method functioned well to isolate the leukocyte nuclei from a blood smear image with much better UR (Under Segmentation Rate), ER (Overall Error Rate), and RDE (Relative Distance Error). The presented LC (Lobe Counting) method is capable of splitting leukocyte nuclei into lobes. The experimental results illuminated that both methods can give expressive performances. In addition, three advanced image processing techniques were proposed as weighted Sobel operator, GDW (Gradient Direction Weight), and GBPD (Genetic-based Parameter Detector).

## Background

Leukocytes, derived from bone marrow stem cells, are the first line of defense of the immune system. Neutrophils, basophils, and eosinophils are called granulocytes because they have granules in their cytoplasm. The other two leukocyte categories, lymphocytes and monocytes, belong to the mononuclear cell group. This means their nucleus is a single piece. These cells are colorless, but they can be colored with special stains to make them visible under the microscope.

**Figure 1:**
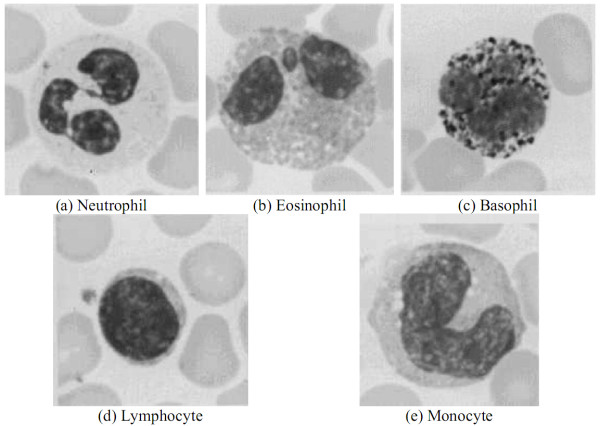
**The micrographs of the five different leukocytes**.

The characteristics of the five leukocyte categories are described as follows. Figure 1 shows the micrographic images of the five different leukocytes [1,2].

### Neutrophil

This granulocyte has very tiny stained granules with low visibility. The nucleus is frequently multi-lobed with lobes connected by thin strands of nuclear material. These cells are capable of phagocytizing foreign cells, toxins, and viruses. This type of cell is the most commonly found, accounting for ***50-70% ***of all leukocytes. If the count exceeds this amount, it is usually caused by an acute infection such as appendicitis, smallpox, or rheumatic fever. If the count is significantly below normal levels, it may be attributed to a viral infection such as influenza, hepatitis, or rubella.

### Eosinophils

This granulocyte has large granules that are acidophilic and appear pink (or red) after staining. The nucleus often has two lobes connected by a band of nuclear material. The granules contain digestive enzymes that are particularly effective against parasitic worms in their larval form. These cells also phagocytize antigen-antibody complexes. Less than ***5% ***of leukocytes are Eosinophils. The increased amount beyond that may be due to parasitic diseases, bronchial asthma, or hay fever. Eosinopenia may occur when the human body is severely stressed.

### Basophil

The basophilic granules in this cell are large, stained deep blue to purple, and are often so numerous that they mask the nucleus. These granules contain histamines (causing vasodilation) and heparin (anticoagulant). They represent less than ***1% ***of all leukocytes. If the count shows an abnormally high number of these cells, hemolytic anemia or chicken pox may be the cause.

### Lymphocyte

The lymphocyte is an agranular cell with a very clear cytoplasm that is pale blue when stained. This cell is much smaller than the three previous granulocytes that are all about the same size. The nucleus of the lymphocyte is stained dark purple and almost fills the cell leaving a very thin rim of cytoplasm. The T-lymphocytes fight against virus infecting cells and tumor cells. The B-lymphocytes, which make up 25-35% of leukocytes, produce antibodies. When there is an overexpression of B-lymphocytes, there may be an infectious mononucleosis or a chronic infection. AIDS patients are required to keep a careful watch on their T-cell level, an indicator of the AIDS virus' activity.

### Monocyte

Agranular in shape, this cell type is the largest among the leukocytes. The nucleus is most often "U" or kidney bean shaped and the cytoplasm is abundant and light blue (bluer than the micrograph illustrates). These cells leave the blood stream (diapedesis) to become macrophages. As a monocyte or macrophage, these cells are phagocytic and defend the body against viruses and bacteria. ***3% ***to ***9% ***of leukocytes are composed of this type of cells. People suffering from malaria, endocarditis, typhoid fever, and Rocky Mountain spotted fever will exhibit an increase the number of monocytes.

High leukocyte counts may be due to inflammation, an immune response, or blood diseases. [3,4]

• An increased percentage of neutrophils may result from:

acute infection, eclampsia, gout, myelocytic leukemia, rheumatoid arthritis, rheumatic fever, acute stress, thyroiditis, or trauma.

• Decreased percentage of neutrophils could be caused by:

aplastic anemia, chemotherapy, influenza, widespread bacterial infection, or radiation therapy or exposure.

• Increasing percentage of lymphocytes may be attributed to:

chronic bacterial infection, infectious hepatitis, infectious mononucleosis, lymphocytic leukemia, multiple myeloma, infectious mononucleosis, mumps, measles, or recovery from a bacterial infection.

• Decreased percentage of lymphocytes may be related to:

chemotherapy, HIV infection, leukemia, radiation therapy or exposure, or sepsis.

• Increased monocytes could result from:

chronic inflammatory disease, parasitic infection, tuberculosis, infectious mononucleosis, mumps, or measles.

• Increased percentage of eosinophils may be caused by:

allergic reaction, cancer, parasitic infection, or Hodgkin's disease.

• Basophils percentage reduction may be due to acute allergic reaction.

Microscopic leukocyte analysis is very useful for identifying or diagnosing many types of diseases [4]. One can recognize the five different leukocytes via their cytoplasmic granules, staining properties of the granules, sizes and shapes of cells, the proportion of nuclear to cytoplasmic material, and the type of nucleolar lobes. Therefore, developing an automatic leukocyte recognition system is feasible via image processing and pattern recognition techniques. It is essential to extract the leukocyte image region from a blood smear image for an automatic leukocyte recognition system. One of the purposes of this paper is to develop an automatic leukocyte nuclei image segmentation method.

A normal neutrophil granulocyte is characterized by the number of nuclear lobes (segments) in the range of two to five. Normally, ***10% ***to ***30% ***of segmented neutrophils have two lobes; the three-lobe type contributes to ***40% ***to ***50%***, and ***10% ***to ***20% ***are four-lobe type. Five-lobe type constitutes of less than ***5%***. When the number of segments is increased to six or more, the cell is hypersegmented. Hypersegmentation is seen most frequently in neutrophils but can also occur in eosinophils and basophils. Hypersegmentation generally represents aging of the cell in the circulation. Corticosteroids usually reduce neutrophil diapedesis into tissues. As a result, neutrophils remain longer in circulation and may partially become hypersegmented. A so-called Neutrophil Right Shift (that is, the number of lobes increases), occurs in the cases of leukemias, chronic nephritis, liver diseases, cancer, sepsis, and vitamin B12 or folate deficiency. Neutrophil hypersegmentation thus has clinically been widely used as an indicator of B12 or folate deficiency. There were many attempts made to quantify the neutrophil right shift [5]. Hence, the other goal of this paper is to develop the automatic method of counting the number of lobes in a cell nucleus. The experimental results show that the proposed methods resulted in impressive segmentation accuracy.

## Related Works

This section will briefly review some techniques used in this paper as well as some cell segmentation methods. In this paper, we will compare their performances with the performance of the method provided in this paper by experiments.

### Mathematical Morphology

Two basic morphological operations for image shape recognition, dilation, and erosion are introduced in this subsection [6]. Erosion can make the objects in a binary image shrink or become thinner. Given an image ***I ***⊆ **Z**^2 ^and a structuring element ***S ***⊆ **Z**^2 ^, erosion shrinks objects by etching away their boundaries. The erosion operation **⊙ **is defined as

(1) $$I⊙S=\{x\in Z^{2}|\forall s\in S,x+s\in I\}.$$

A binary image contains only two colors: background color and foreground color respectively, described by a ***0***-bit and a ***1***-bit. Dilation allows objects' images to expand, thus potentially filling in small holes and connecting disjointed objects. The dilation operation can be defined as the following:

(2) $$I⊕S=\{c\in Z^{2}|\exists i\in I,\exists s\in S,c=i+s\}$$

### Cell Segmentation Methods

Four cell segmentation methods are reviewed: Bone Marrow Leukocyte Segmentation (BMLS) method [7], Nuclei Position Detection (NPD) method [8], Fuzzy-based Cell Detection (FCD) method [9], and Color and Active Contour based Detection (CACD) [10]. Their performances will be compared to the segmentation method proposed in this paper.

The BMLS method [7] was to analyze a set of leukocyte-nucleus-based features using mathematical morphology. It applies the opening operation [6] on an image and increases the size of the structuring element in order to diminish the objects on the image.

The NPD method was developed to automatically segment the cells from genome-wide RNAi (RNA interference) screening images. The nuclei can be separated from DNA channel by using a modified watershed algorithm. The images of cells were then extracted by modeling the interaction between the cells, and by combining both gradient and region information in the Actin and Rac channels. A new energy function was formulated based on an interaction model for segmenting tightly clustered cells with significant intensity variance and specific phenotypes, and minimized by using a multiphase level set method. In NPD, Otsu's threshold method is first applied to determine a threshold ***T_c _***to classify all the pixels into two classes. The distance transform was employed to calculate the shortest distance between each pixel to the non-zero pixel. Finally, the watershed transform was employed to segment the contours of all objects in the image.

The FCD method [11] was to track neural stem cells in a sequence of images. Users can interactively verify and correct the crucial starting segmentation of the first frame, and also inspect the final result while correcting errors if necessary. All cells are classified into inactive, active, dividing, and clustered cells. Different algorithms are employed to deal with different cell categories. A special backtracking step was used to automatically correct some common errors that appear in the initial forward tracking process. The fuzzy threshold method was first applied to classify all the pixels of an image. Two thresholds were calculated. All pixels with grey-level intensity below the lower threshold were set to ***0 ***and all pixels above the higher threshold were set to ***1***. The gray-level intensities of the remaining pixels, whose gray-level intensities lie between the lower threshold and the higher threshold, are linearly rescaled to the range **[*0*, *1*]**. Then the distance transformed is applied to calculate the shortest distance of any one pixel to the non-zero pixel. Finally, the maximum transform and watershed transform will be applied to determine the contours of all objects in the image.

The CACD method [7] was to cut off the leukocytes from a color blood smear image. In this method, Otsu's threshold method was used to determine a threshold on the green channel of the image. Via the threshold, the initial contours of nucleuses can be detected from the image. Based on the initial contour, active contour method was employed to find the precise boundaries of cytoplasm.

### Error Measure of Segmentation

In this paper, four segmentation error measures were used to evaluate the performance of a segmentation method. Over-segmentation rate (***OR***), Under- segmentation Rate (***UR***), and Overall Error Rate (***ER***) are often applied to evaluate the ability of a segmentation method in severing the ROI (Region Of Interest) from an image [12]. Let ***O_p _***be the number of object pixels in the segmentation results but actually not, ***U_P _***be the number of pixels not in the segmentation result but actually included, and ***D_P _***be the number of pixels in the segmentation result and actually included. ***OR***, ***UR ***and ***ER ***can be described as:

(3) $$OR=\frac{O_{p}}{U_{p}+D_{p}}$$

(4) $$UR=\frac{U_{p}}{U_{p}+D_{p}},$$

and

(5) $$ER=\frac{O_{p}+U_{p}}{D_{p}}.$$

Yang-Mao *et al*. [13] proposed RDE (Relative Distance Error) to evaluate object segment results. Let $e_{1},e_{2},...,e_{n_{e}}$ be the pixels on ***E***, and let $t_{1},t_{2},...,t_{n_{t}}$ be the pixels on ***T***, where ***E ***and ***T ***are respectively the contour pixels on the segmented object and the ground truth object, and ***n_e _***as well as ***n_t _***are the number of pixels on ***E ***and ***T***, respectively. RDE is defined as:

(6) $$RDE=\frac{1}{2}\left(\sqrt{\frac{1}{n_{e}}\sum_{i=1}^{n_{e}}d_{e_{i}}^{2}}+\sqrt{\frac{1}{n_{t}}\sum_{i=1}^{n_{t}}d_{t_{i}}^{2}}\right),$$

where $d_{e_{i}}$**= *min*{distance(*e_i_, t_j_*)|*j *= *1,2*, ..., *n_t_*},**

$d_{t_{j}}$**= *min*{distance(*e_i_, t_j_*)|*i *= *1,2*, ..., *n_e_*}**, and

**distance(*e_i_, t_j_*) **is the Euclidean distance between ***e_i _***and ***t_j_***.

## Results

The purpose of this section is to investigate the performances of the LNS method in leukocyte nuclei segmentation and the LC method in lobe counting by experiments. In order to verify the adaptability of the LNS method, two image sets are used as the test data. The two image sets are obtained from different laboratories and different equipments. There are ***29 ***images in set 1 (provided by Prof. Meng-Hsiun Tsai, Dartpartment of Information Systems, National Chung Hsing Universtiy) and ***41 ***images in set 2 (provided by Dr. Guo-Qing Liu, Department of Medical Laboratory Science and Biotechnology, China Medical University). Totally, there are ***47 ***leukocytes on all the images in set 1 and ***53 ***leukocytes on all the images in set 2. These images were taken with optic microscopes at about ***800 ***to ***1000 ***times magnification. The contours of the leukocyte nuclei manually drawn by a biologist are served as the ground truth. Four of the test images are randomly selected to train the most suitable ***r_s _= 0.6***, ***r_G _= 2.5***, ***r_t _= 0.8***, ***r_r _= 0.7***, ***t_1 _*= *23***, ***t_2 _*= *352***, ***t_3 _*= *25***, ***t_4 _*= *830***, ***t_5 _*= *0.1***, and ***t_6 _*= *0.1 ***via GBPD, where the parameters used are given to $n_{r_{1}}$***= 10***, $n_{r_{2}}$***= 16***, $n_{r_{3}}$***= 10***, $n_{r_{4}}$***= 10***, ***N = 10***, ***N' = 10***, and ***n_1 _***= ***n_2 _***= ***n_3 _***= ***n_4 _***= ***n_5 _***= ***n_6 _= 40***, and the lobes in the test images were counted by the biologist in advance. **MAX_#EROSION **is set to ***20***.

**Figure 2:**
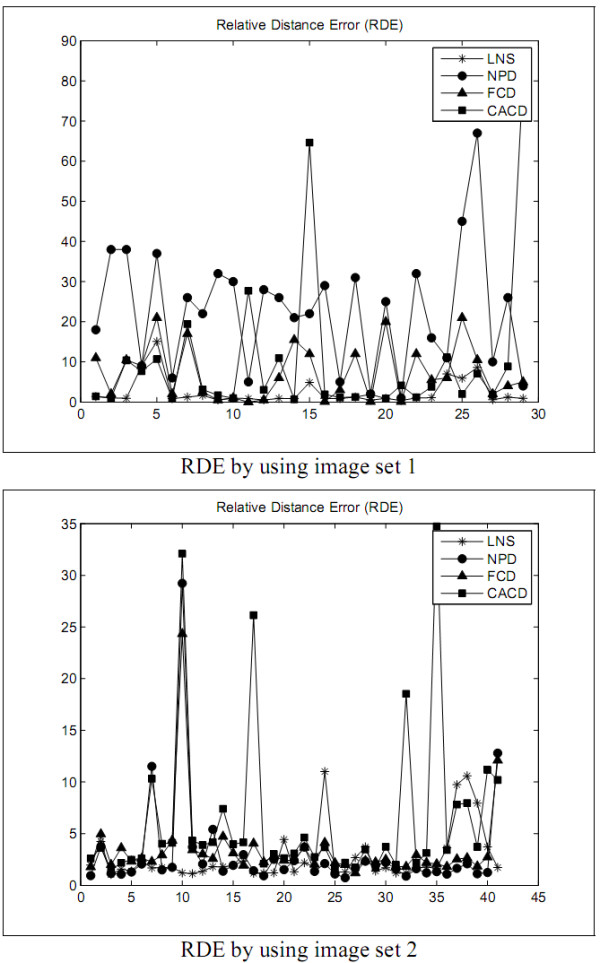
**The ***RDE ***of the first experiment**.

**Figure 3:**
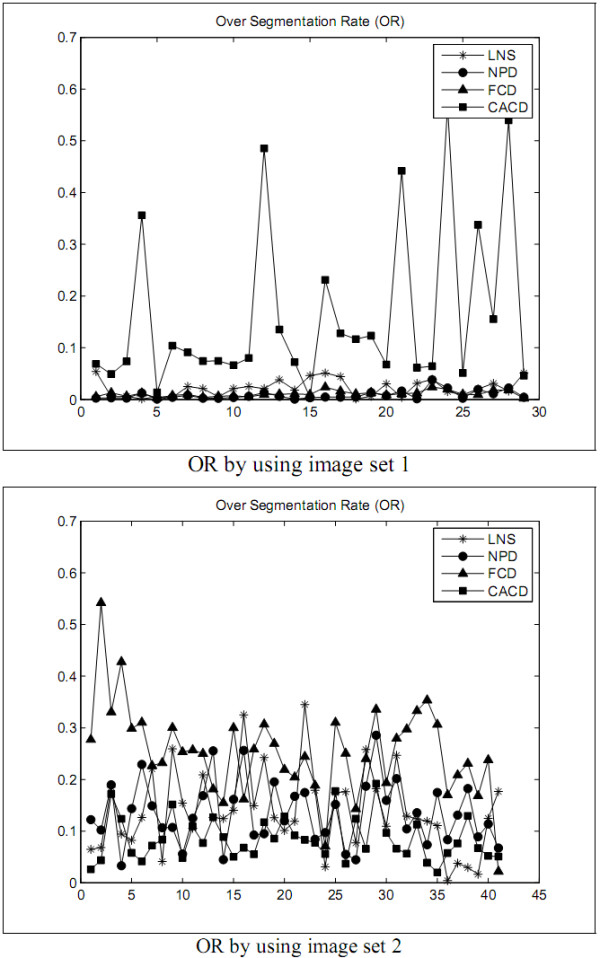
**The ***OR ***of the first experiment**.

**Figure 4:**
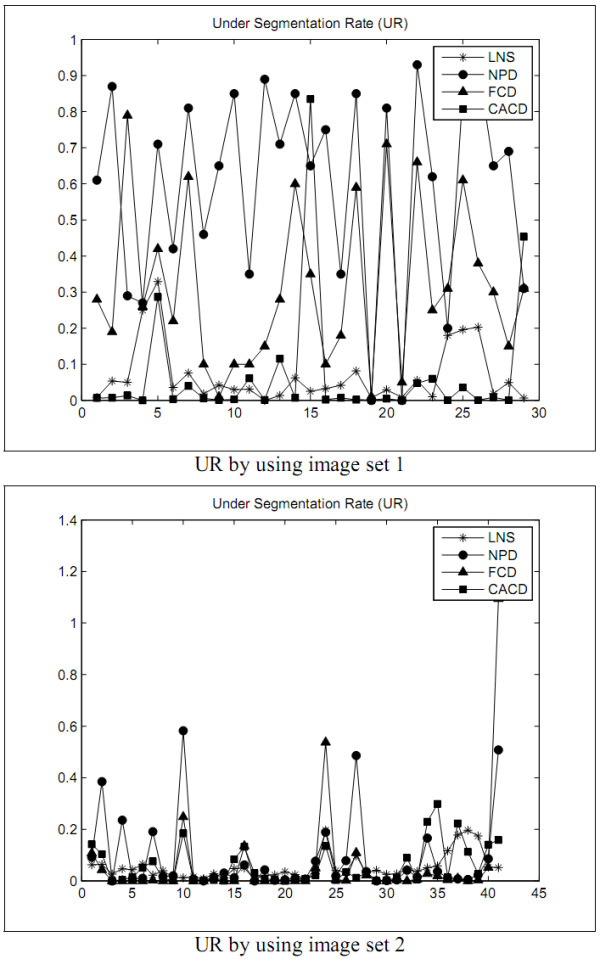
**The ***UR ***of the first experiment**.

**Figure 5:**
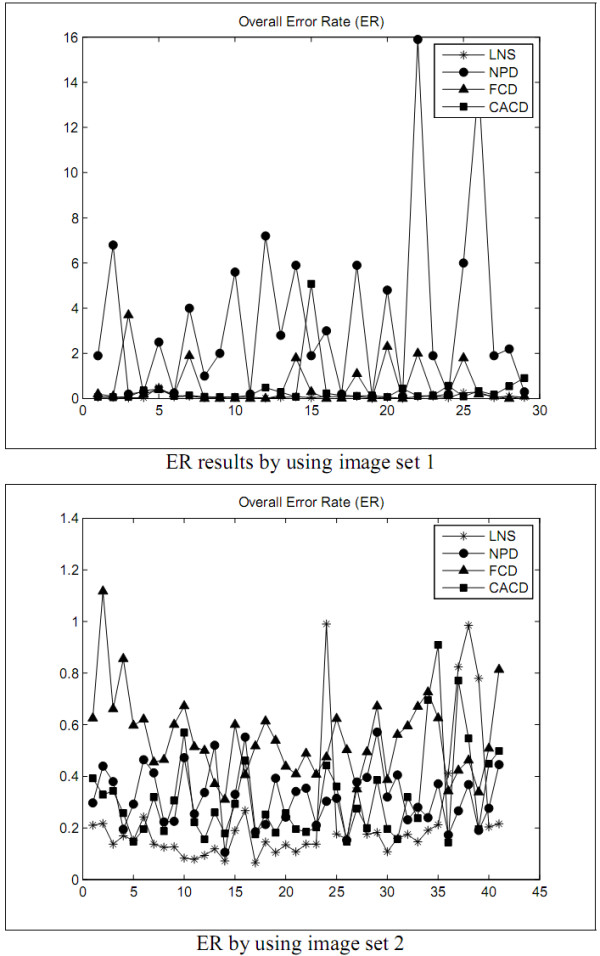
**The ***ER ***of the first experiment**.

The first experiment is designed to explore the performance of the LNS method and to compare with the performances of the NPD, FCD, and CACD methods in segmenting leukocyte nuclei out from a blood smear image. The segmentation errors are shown in Figures 2, 3, 4, 5 and Tables 1 and 2. The experimental results illustrate that the LNS method produces much better ***UR***, ***ER***, and ***RDE ***than the NPD, FCD, and CACD methods by using the images in sets 1 and 2 as the test images.

**Table 1:** The average segmentation errors by using the images in set 1 of test images

Error/Method	***OR***	***UR***	***ER***	***RDE***
LNS	***0.028***	***0.064***	***0.105***	***2.080***
NPD	***0.008***	***0.596***	***3.379***	***22.772***
FCD	***0.009***	***0.317***	***0.737***	***8.091***
CACD	***0.161***	***0.694***	***0.398***	***10.068***

**Table 2:** The average segmentation errors by using the images in set 2 of test images

Error/Method	***OR***	***UR***	***ER***	***RDE***
LNS	***0.134***	***0.051***	***0.23***	***2.683***
NPD	***0.134***	***0.086***	***0.32***	***3.029***
FCD	***0.252***	***0.061***	***0.545***	***3.432***
CACD	***0.084***	***0.059***	***0.317***	***6.354***

**Figure 6:**
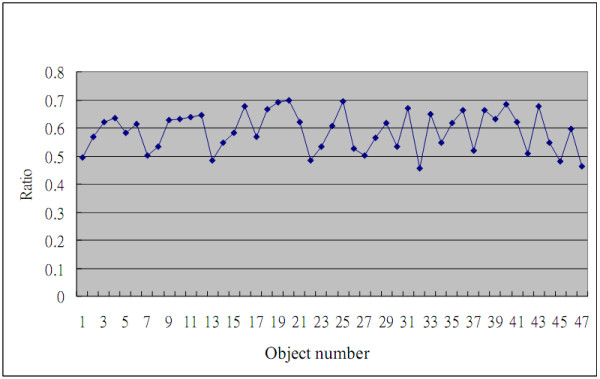
**The area ratio ***R ***of a nucleus to the related MRC**.

The second experiment is designed to scrutinize the performance of the LC method in splitting the leukocyte nuclei into lobes. The LC method is used to detect whether a leukocyte nucleus comprises more than one lobe or not, and then to separate those seemed-multi-lobe image of the nucleus into clear lobes. If the area ratio ***R ***of the leukocyte nucleus to its MBR is less than a threshold ***r_A_***, the leukocyte nucleus is considered to be the nucleus containing more than one lobe. The ***R***'s of ***47 ***leukocyte nuclei to their MBRs is shown in Figure 6, where the ***47 ***leukocyte nuclei have to be split further. The curve in Figure 6 displays that its ***R ***is almost less than ***0.7 ***for each leukocyte nuclei. Therefore, in this experiment, ***r_A _***is set to ***0.7***.

The LC method is used to split the leukocyte nuclei into lobes with ***r_A _*= *0.7***. In this experiment, the biological expert figures out ***223 ***leukocyte nucleus lobes in the ***29 ***test images. The leukocyte nuclei were split into lobes and counted that there are ***186 ***leukocyte nucleus lobes in the ***29 ***test images. The accuracy rate of ***83.41% ***resulted from counting the leukocyte nucleus lobes on the blood smear images by the LC method.

## Discussion

The first experimental results show that the LNS method is inferior to the NPD and FCD methods but worse than the CACD method in ***OR ***by using the images in set 1 as test data. With set 2 as test data, the LNS method performed better ***OR ***than the CACD method but worse than the FCD method, and as excellently as the NPD method. The results of this experiment revealed that the LNS method resulted in much better ***UR***, ***ER***, and ***RDE ***and is much less sensitive to the variation of images.

**Figure 7:**
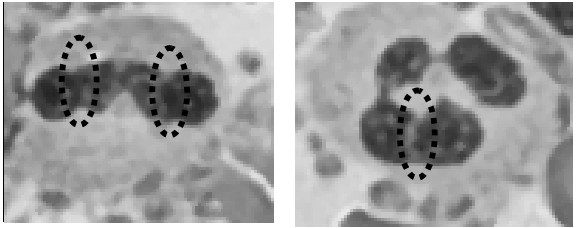
**The white blood cell with obscure fracture**.

In the primary stage of a continued "right shift" (increasing the number of lobes), a leukocyte nucleus was twisted and slightly indented, such as the regions indicated by the black dashes in Figure 7. The experimental results show that LC method can provide a good lobe split for most leukocyte nuclei, except the leukocyte nuclei with a slight indentation.

## Conclusions

Insensitive to the variance of images, the LNS method functioned well to isolate the leukocyte nuclei from a blood smear image with much better ***UR***, ***ER***, and ***RDE***. The presented LC method is capable of splitting leukocyte nuclei into lobes. The experimental results illuminated that both methods can give expressive performances. In addition, three advanced image processing techniques were proposed as weighted Sobel operator, GDW, and GBPD. In a weighted Sobel operator, a user can give the most suitable ***r_s _***to satisfy his requirement. A bigger ***r_s _***is required for the user to accentuate the objects with a more definite contour. To highlight the objects with an indistinct contour, a smaller ***r_s _***has to be assigned. GDW can not only enhance the object's contour, but also suppress the noise's contour. GBPD was used to determine the optimal parameters that were used in LNS method.

## Methods

In this study, a Leukocyte Nuclei Segmentation (LNS) method was proposed to automatically extract the leukocyte nuclei region from a blood smear image. The LNS method consists of two stages: Object Contour Detection and Leukocyte Nuclei Segmentation. A blood smear image is the image mixture of possible leukocytes, erythrocyte cells, platelets, leukocyte nuclei, and noise. The goal of the object contour detection stage is to locate all the objects on the image. At the leukocyte nuclei segmentation stage, leukocyte nucleus objects were then filtered out based on the gray-level intensities and the sizes of the objects obtained at the object contour detection stage.

### Object Contour Detection

**Figure 8:**
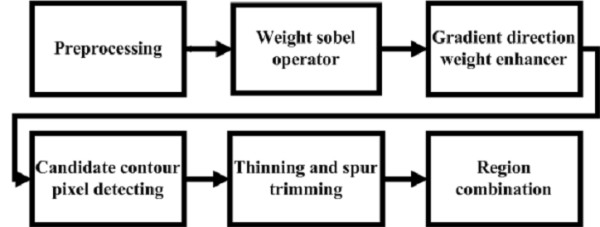
**The flowchart of leukocyte nucleus segmentation processing**.

During the object contour detection stage, there are six approaches: preprocessing, weighted Sobel operator, gradient direction weight enhancer, candidate contour pixel detecting, thinning and spur trimming, and region combination. The flowchart of LNS method is shown in Figure 8; this section will introduce each of these approaches in detail.

### Preprocessing

The blood smear may be stained by different color dyes. To avoid being influenced by dye color, all blood smear images were first transformed into gray-level. In order to diminish the variation of images, the pixels' gray-level intensities of a blood smear image ***I_0 _***were then stretched to the full ***0 ***to ***255 ***range. Let ***I_0_*(*x, y*) **(resp. ***I_p_*(*x, y*)**) be the pixel located at the coordinates **(*x, y*) **on ***I_0 _***(resp. ***I_p_***), and ***max ***as well as ***min ***the maximal and the minimal gray-level intensities of all the pixels in ***I_0_***_, _respectively. ***I_0 _***is then transformed into ***I_p _***by $I_{p}(x,y)=255\times \left(\frac{I_{0}\text{(}x,y\text{)}-min}{max-min}\right)$ to reduce the variation among all different images.

### Weighted Sobel Operator

**Figure 9:**
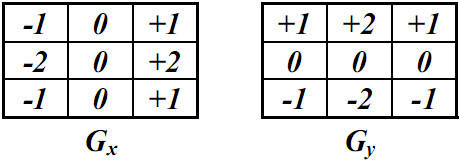
**Two convolution masks of Sobel operator**.

An edge generally corresponds to a set of strong illumination gradients. Sobel operator [14] is one of the simplest and most effective gradient computation methods. LNS method will employ Sobel operator to calculate the gradients of the pixels in ***I_p_***. Two ***3×3 ***convolution masks shown in Figure 9 are employed in Sobel operator. We call ***W*(*x*, *y*) **a corresponding window of ***I_p_*(*x, y*) **where ***I_p_*(*x, y*) **is located at the center of ***W*(*x, y*) **and ***W*(*x, y*) **consists of ***m*×*m ***pixels. ***I_p_*(*x *± *x'*, *y *± *y'*) **are the pixels in ***W*(*x, y*) **for $0\leq x'\leq \frac{m}{2}$ and $0\leq y'\leq \frac{m}{2}$.

Let the corresponding window ***W*(*x*, *y*) **of ***I_p_*(*x, y*) **consist of ***3 × 3 ***pixels. The Sobel operator defines the gradient ***g*(*x*, *y*) **of ***I_p_*(*x*, *y*) **as the following:

(7) $$\Delta G_{x}(x,y)=G_{x}⊗W_{w}(x,y),$$

(8) $$\Delta G_{y}(x,y)=G_{y}⊗W_{w}(x,y),$$

and

(9) $$g(x,y)={\left(\Delta G_{x}{}^{2}(x,y)+\Delta G_{y}{}^{2}(x,y)\right)}^{1/2},$$

where **⊗ **is the operator of convolution.

Different users prefer either to highlight the gradients of the pixels with high gradients, or to enhance the gradients of the pixels with low gradients. To solve these problem caused by human preference, a Weighted Sobel Operator (WSO) was then proposed by the authors. Let ***g_M _***and ***g_m _***be the maximal and minimal ones of all the pixel gradients in ***I_p_***. This weighted sobel operator assigns $g(x,y)=255\times {\left(\frac{g\text{(}x,y\text{)}-g_{m}}{g_{M}-g_{m}}\right)}^{r_{s}}$ to the gray-level intensity of the pixel ***I_g_*(*x*, *y*) **located at the coordinates **(*x*, *y*) **in ***I_g_***, where ***r_s _***is a given constant. Hence, ***I_g _***can be a gray-level image regarding the gradients of the pixels in ***I_p_***.

**Figure 10:**
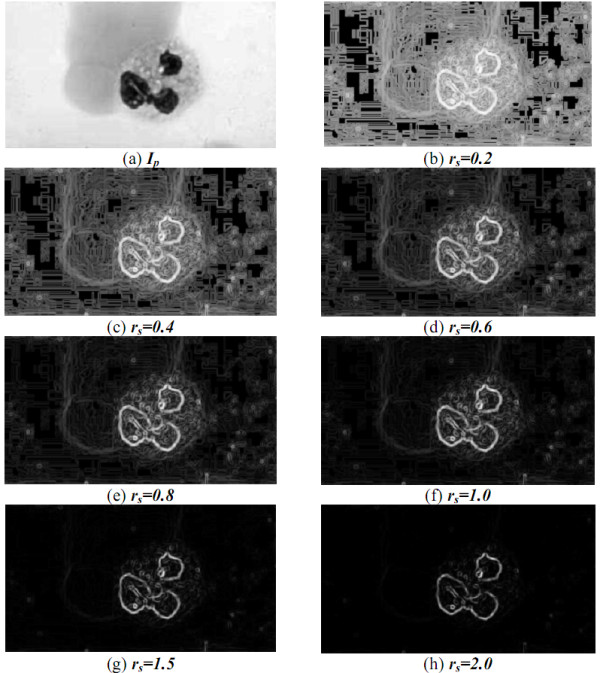
**The gradients obtained by the weighted Sobel operator with different ***r_s_*****.

Given a big ***r_s _***(i.e. ***r_s_>1***), WSO will enhance the pixel with a high gradient but suppress the pixel with low gradient obtained by Sobel operator. Contrarily, when given a small ***r_s _***(i.e. ***r_s_*<*1***), it will inhibit the pixel with high gradient but highlight the pixel with low gradient computed by Sobel operator. The gradients obtained by the weighted Sobel operator with different ***r_s _***are shown in Figure 10. A generic algorithm was then used to decide the optimal value of ***r_s _***later.

### Gradient Direction Weight Enhancer

**Figure 11:**
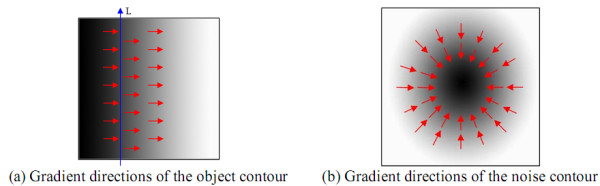
**The difference of contour gradient directions of objects and noises**.

Given a smaller ***r_s_***, the weighted Sobel operator can make the object contour more obvious but also raising the gradient of noise. A GDW (Gradient Direction Weight) Enhancer was proposed in this paper to lower the gradient of noise contour and enhance the gradient of object contour. The gradient directions of the pixels near the object contour are usually almost perpendicular to the direction of the object contour. In microscopic viewpoints, a small object contour segment is close to one straight line. For example in Figure 11(a), the line ***L ***is an object contour segment and the arrows are the gradient directions of the pixels near the object contour segment. Moreover, these gradient directions near a noise contour are shown in Figure 11(b). Based on this property, a GDW enhancer was proposed to simultaneously brighten the gradient of the object contour and to suppress the gradient of the noise contour.

**Figure 12:**
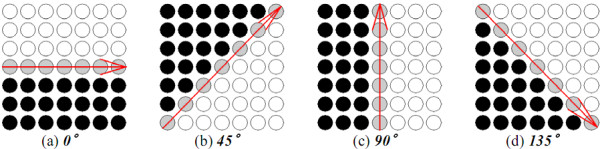
**Four ***θ_L_***-partitions of ***W*****.

The gradient direction of a pixel ***I_0_*(*x*, *y*) **can be defined as $\theta_{g}=tan^{-1}\left[\frac{\text{Δ}G_{y}\text{(}x,y\text{)}}{\text{Δ}G_{x}\text{(}x,y\text{)}}\right]$, where **Δ*G_x_*(*x*, *y*) **and **Δ*G_y_*(*x*, *y*) **can be computed by Formula (7) and (8). Assume that one object contour segment ***L ***passes through ***I_p_*(*x*, *y*)**, and the angle of ***L ***inclined to the horizontal axis is ***θ_L_***. The GDW enhancer first estimates ***θ_L_***, which is supposed to be close to one of ***0*°**, ***45*°**, ***90*°**, and ***135*°**. Let ***W_G _***be a corresponding window of ***I_p_*(*x*, *y*) **composed of ***m_G_*×*m_G _***pixels and divided into two equal regions according to the four different possible directions of ***L ***at angles ***0*°**, ***45*°**, ***90*°**, and ***135*° **with respect to the horizontal line. Four different partitions where ***m_G _= 7 ***are shown in Figure 12. The black dots and the white dots signify the black region and the white region, respectively. The partitions in Figure 12 was named ***θ**-*partitions for ***θ = 0°***, ***45°***, ***90°***, and ***135°***, respectively. For each ***θ**-*partition, ***d_θ _*= |*c_b_*-*c_w_*| **was calculated, where ***c_b _***and ***c_w _***are the average gray-level intensities of the black and white regions, respectively. Here, the estimated angle ***θ_L _***of ***L ***is defined as $\theta_{L}=arg\left(\underset{\theta =0{}^{\circ},45{}^{\circ},90{}^{\circ},135{}^{\circ}}{\max}\left(d_{\theta }\right)\right)$ and the GDW of ***I_p_*(*x*, *y*) **as **|*sin*(*θ_L_-θ_g_*)|**.

**Figure 13:**
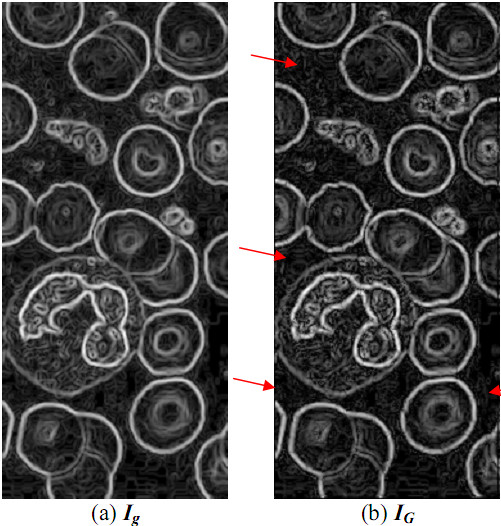
**The images before and after processed by GDW**.

As **|*θ_L_-θ_g_*| **is closer to ***90° ***and ***I_g_*(*x*, *y*) **is bigger, the probability of ***I_0_*(*x*, *y*) **located at the object contour is higher. Therefore, with the GDW enhancer, $I_{g}(x,y)\;\times |\sin\text{(}\theta_{L}\text{-}\theta_{g}\text{)}{|}^{r_{G}}$ can be assigned to the gray-level intensity of ***I_G_*(*x*, *y*) **for generating a new gray-level image ***I_G_***, where ***r_G _***is a given constant. The optimal constant ***r_G _***will be decided by a genetic algorithm. The images before and after the GDW enhancer processing are shown in Figure 13.

### Candidate Contour Pixel Detecting

The gray-level intensity of ***I_G_*(*x*, *y*) **represents the possibility of ***I_0_*(*x*, *y*) **to be an object contour pixel. To successfully cut off objects from ***I_0 _***following GDW enhancer approach processing, given an adaptive threshold to isolate the possible object contour pixels is a pre-requisite. Given a bigger threshold, higher contrast edges may be detected but some desired edges with low contrast may be overlooked. On the contrary, lower contrast edges may be gleamed given a smaller threshold, but more noise edges may probably be collected simultaneously. One of the commonly used threshold decision making methods, Otsu's method [15], is thus utilized in LNS method to specify the threshold ***Th***. Otsu's method exhaustively searches for the threshold t* that minimizes the within-class variance, defined as a weighted sum of variances of two classes:

(10) $$t*=Arg\text{(}\underset{0\leq t<L}{Min}\text{(}p_{1}\text{(}t\text{)}\sigma_{1}^{2}\text{(}t\text{)}+p_{2}\text{(}t\text{)}\sigma_{2}^{2}\text{(}t\text{)))},$$

where weight ***p_i _***is the probability of a pixel in the ***i***-th class separated by a threshold ***t ***and $\sigma_{i}^{2}$ the variance of pixels' gray-level intensities in the ***i***-th classes.

**Figure 14:**
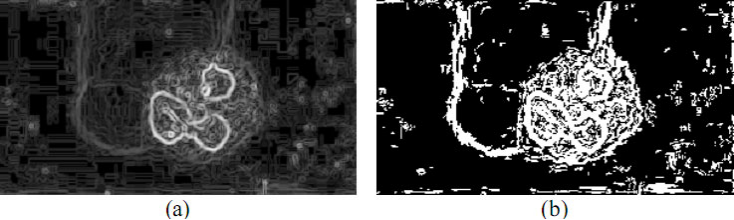
*****I_G _***and its corresponding ***I_b_*****.

In LNS method, each pixel ***I_G_*(*x*, *y*) **in ***I_G _***was swept to generate a binary image ***I_b_***. The threshold t* was then applied to obtain an appropriate threshold t*×*r_t _*for better candidate object contour extraction. The optimal ***r_t _***couldbe obtained by a genetic algorithm which will be introduced later in this paper. If ***I_G_*(*x*, *y*) **is greater than or equal to t*×*r_t_*, then ***1 ***was assigned to ***I_b_*(*x*, *y*)**; otherwise, a value of ***0 ***would be assigned. The pixel ***I_b_*(*x*, *y*) **with ***1***-bit is called a candidate object contour pixel. One ***I_G _***and its corresponding ***I_b _***are demonstrated in Figure 14.

### Thinning and Spur Trimming

Noises in an image or the exquisite vein, and the pixels at the vicinity of object contour may cause false edges. The expected thickness of an object contour would be one-pixel. In this paper, the Hit-and-Miss Transform-based Skeletonization (HMTS) algorithm [6] is used to reduce the object contour in the thickness of one pixel. The eliminated candidate edge pixels were named as redundant-edge pixels and the remaining candidate edge pixels as true-edge pixels by the authors.

**Figure 15:**
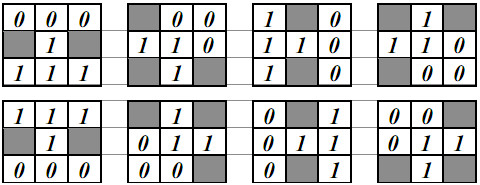
**Eight structuring elements for thinning**.

**Figure 16:**
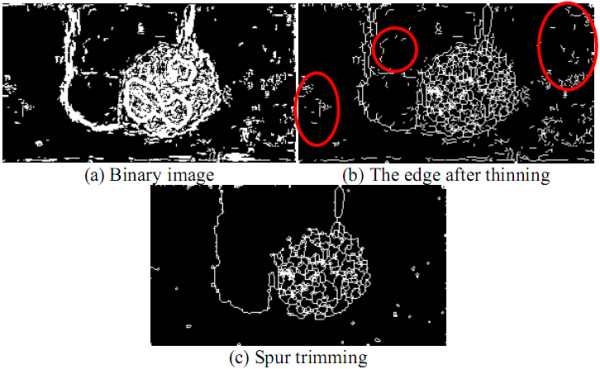
**The results after thinning and trimming spurs**.

Thereafter, the region detecting approach takes the HMTS algorithm [12,14] to reduce the object contour thickness in one pixel. Let each pixel ***I_b_*(*x*, *y*) **in ***I_b _***correspond to a window ***W_t_*(*x*, *y*)**, where ***W_t_*(*x*, *y*) **consists of ***3×3 ***pixels and ***I_b_*(*x*, *y*) **is the central pixel of ***W_t_*(*x*, *y*)**. ***W_t_*(*x*, *y*) **was compared with each of the eight structuring elements in HMTS algorithm shown in Figure 15, where the gray pixels stand for the don't-care pixels (A don't-care pixel may be a ***1***-bit pixel or a ***0***-bit pixel). ***W_t_*(*x*, *y*) **is matched if the positions and values of ***1***- and ***0***-bits on one structuring element are completely the same as those on ***W_t_*(*x*, *y*)**, regardless of don't-care pixels. When ***W_t_*(*x*, *y*) **is matched, ***I_b_*(*x*, *y*) **is changed into ***0***. This procedure was repeated until no more thinning needs to be performed in this algorithm. The HMTS algorithm has been performed to cut off the redundant-edge pixels, resulting in single-pixel edge thickness. The result after running the thinning operation is shown on Figure 16(b).

**Figure 17:**
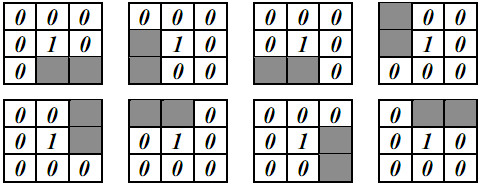
**Eight structuring elements for trimming spurs**.

After being processed by HMTS method, some small spurs may appear on the skeleton, which are not the desired edges. Therefore, a trimming spur algorithm is required to remove the spurs. The procedure of the trimming spur algorithm [13] is exactly the same as that of above thinning algorithm except for the eight structuring elements in Figure 15, which are replaced by those in Figure 17. The result obtained by the trimming spur algorithm on the image in Figure 16(b) is shown in Figure 16(c). Let ***I_e _***be the binary contour image, which has been processed by the trimming spur algorithm.

### Region Combination

Since the cytoplasm and nuclei of leukocytes are frequently uneven, a nucleus may be segmented into several small regions after the previous image processing. In addition, some noises may still exist on the blood smear image. Therefore, some regions in ***I_e _***must be combined into one or be removed. Let ***B ***and ***B' ***be two different regions on ***I_p_***, the contours of which are marked on ***I_e_***, where ***B' ***is adjoining to ***B ***and ***B' ***has the minimal gray-level intensity difference from ***B***.

**Figure 18:**
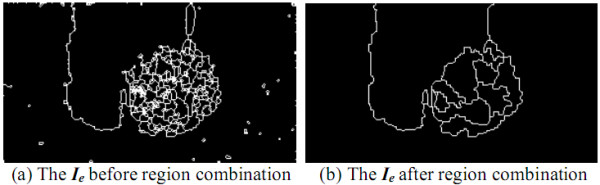
**The result after region combination**.

Let ***A ***and ***A' ***be the numbers of pixels in ***B ***and ***B' ***respectively, and ***C ***and ***C' ***be the average gray-level intensities of all the pixels in ***B ***and in ***B'*, **respectively. As one of the following criteria is satisfied, ***B ***can be combined to ***B'***:

1) ***A *≤ *t_1_***;

2) ***A *≥ *t_2_***, **|*C ***- ***C'**| ***≤ *t_3_***, and ***B ***is located at the image boundary of ***I_p _***(a part of ***B ***is not in ***I_0_***);

3) ***A *≥ *t_4_***, **|*C ***- ***C'**| ***≤ *t_3_***, and ***B ***is not located at the image boundary of ***I_p_***,

where ***t_1_*, *t_2_*, ..., *t_4 _***are four given thresholds. The result after combining the segmented regions in Figure 18(a) is shown in Figure 18(b).

### Leukocyte Nuclei Segmentation

After the regions combination, every closed curve in ***I_e _***represents an object. There may be many kinds of objects, like erythrocytes, the cytoplasts and nuclei of leukocyte, platelets, and noise in a blood smear image. This stage is intended to filter out the objects of leukocyte nuclei from ***I_e_***. The leukocyte nucleus is usually darker than the other blood cell's. The cytoplasts of basophil and lymphocyte are much darker than those of the erythrocyte cells, and the cytoplasts of other leukocytes are brighter or a little darker than those of the erythrocytes. The area of platelets is much smaller than that of the lymphocyte nuclei. Based on these properties, the leukocyte nuclei can be filtered out.

**Figure 19:**
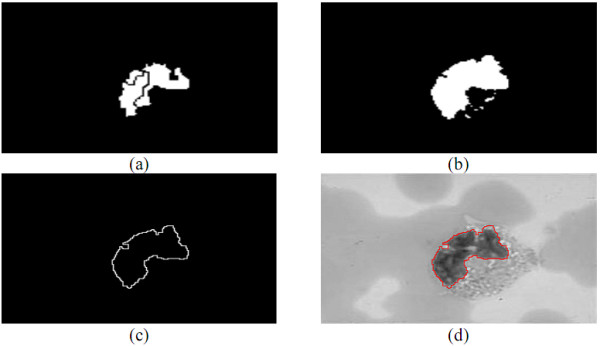
**The segmentation results obtained in the leukocyte nuclei segmentation stage**.

Let ***C_ave _***be the average gray-level intensity of all the pixels in all the objects indicated by ***I_e_***. Since the leukocyte nuclei is darker than others, if the average gray-level intensity of an object is smaller than or equal to ***T = C_ave_×r_r_***, then this object is regarded as a leukocyte nuclei, where ***r_r _***is a given constant. Through the above processing, the filtered-out leukocyte nuclei for ***r_r _*= *0.7 ***is shown in Figure 19(a). This paper will use a genetic algorithm to obtain the optimal ***r_r_***. The textures of leukocyte cytoplasts and nuclei are often uneven. For segmenting the leukocyte nuclei more accurately, this stage refines the contours on ***I_e_***. If a pixel inside a leukocyte nuclei contour in ***I_e _***with gray-level intensity larger than ***C_ave_***, then this pixel is assigned to a non-leukocyte nuclei pixel. If a pixel is outside the leukocyte nuclei contours and the minimal distance between the pixel and the contour indicated in ***I_e _***is less than ***5 ***pixels and its gray-level intensity smaller than or equal to ***T***, the pixel is considered a leukocyte nuclei pixel.

Some objects in ***I_e _***may be platelets or noise, which are generally smaller than the lobes of leukocyte nucleus; the region with a large area in ***I_e _***is always a white blood cell or consists of several erythrocytes overlapping together. According to this property, the segmented objects were sorted according to their sizes. Let ***A_m _***be the area of the median object in size. Sized less than ***t_5_*×*A_m_***, the object was removed by the LNS method. The results after refining the contours on the image in Figure 19(a) are shown in Figure 19(b); the results after removing the small objects are shown in Figure 19(c); Figure 19(d) is the segmented contours.

A leukocyte nucleus probably consists of several nuclei. If the contours of two nuclei are very close, both nuclei are considered the same leukocyte nuclei. As the sizes of two objects are less than ***A_m _***and the distance of two closest pixels between the contours on the two nuclei is less than or equal to $t_{6}\times \sqrt{\frac{A_{m}}{\pi }}$, both leukocyte nuclei are considered the same.

### Lobe Counting

The shape of the leukocyte nucleus is one of the most important features in determining the type of the leukocyte nucleus. The number of lobes can be used to describe the phenomenon of neutrophil right shift. In this section, Lobe Counting (LC) method is presented to count the number of lobes in a leukocyte nucleus indicated in ***I_e_***.

**Figure 20:**
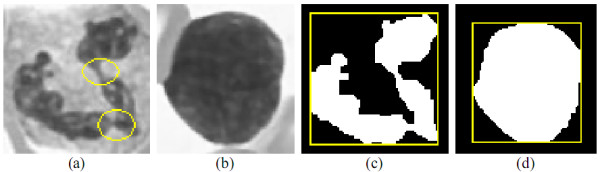
**The leukocyte nuclei and their MBR**.

A leukocyte nucleus can be completely encircled by a Minimum Bounding Rectangle (MBR), shown as Figures 20(c) and 14(d). While the contour of the leukocyte nucleus is very crooked and uneven, the ratio ***R ***of leukocyte nucleus area to its MBR's is small. If ***R ***is less than a threshold ***r_A_***, the nucleus is considered to comprise more than one lobe and their lobes need cutting off, i.e. the nucleus in Figure 20(c); otherwise, the nucleus is considered to contain only one lobe, and it is unnecessary to split the nucleus as shown in Figure 20(d).

*Obj_cut*(*obj*)

(1) While ***obj.#iteration *<MAX_#EROSION**

(2)   Erode object ***obj***

(3)   If ***obj ***will vanish in next erosion then   /* ***obj ***will disappear in next erosion operation */

(4)      Dilate ***obj obj.#iteration ***runs and return the object ***obj***

(5)   If ***obj ***is not split into some sub-objects then

(6)      ***obj.#iteration = obj.#iteration + 1***

(7)      ***Obj_cut*(*obj*)**      /* continue to run erosion operation */

(8)   If ***obj ***is split into sub-objects ***obj_1_***, ***obj_2_***, ..., ***obj_n _***then

(9)      for ***i = 1 ***to ***n***

(10)         ***obj_i_.#iteration = 0***

(11)      ***Obj_cut*(*obj_i_*)**      /* continue to run erosion operation */

(12)         Dilate ***obj_i _obj_i_.#iteration ***runs and return ***obj_i_***

(13) Dilate ***obj obj.#iteration ***runs and return ***obj***

**Figure 21:**
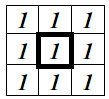
**The structuring element of erosion and dilation operation**.

A junction between two lobes in a leukocyte nucleus is usually at the contour crooked extremely or at the narrow part of leukocyte nucleus, shown in Figure 20(a). Erosion and dilation operations [6] were applied in the LC method to separate the lobes. Let ***obj ***be a nucleus which consists of two parameters, the nucleus of object ***obj ***and ***obj*.*#iteration***. With ***obj*.*#iteration***, the number of iterations ascends to execute erosion then dilation operation. **MAX_#EROSION **is the given maximal number of iterations in eroded ***obj***. Algorithm ***Obj_cut*(*obj*) **functions to cut off the lobes from ***obj ***with the structuring element in Figure 21 for erosion and dilation operations.

In an ***Obj_cut*(*obj*)**, after executing erosion operation on object ***obj***, ***obj ***may disappear, not be split into some sub-objects, or be split into some sub-objects. Lines 3 and 4 handle the case where ***obj ***will disappear in the next erosion operation. Lines (5) to (7) deal with the ***obj ***that is not split into some sub-objects. If ***obj ***is cut into some sub-objects, each sub-object will be split continually. Lines (8) to (12) perform it.

**Figure 22:**
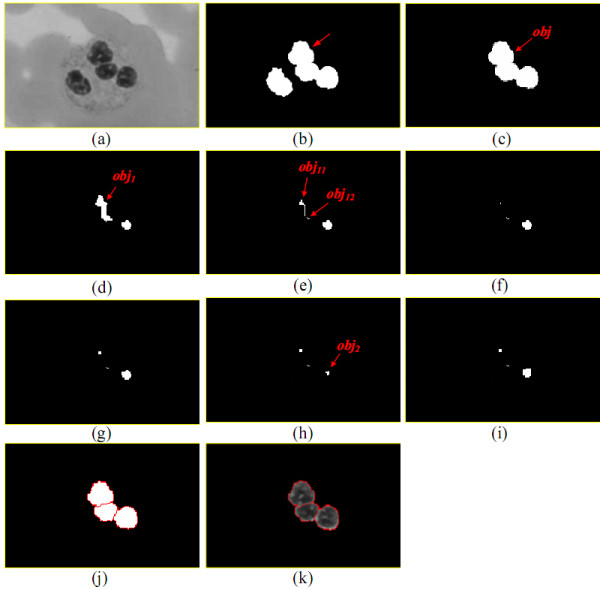
**The procedure severing out the lobes from an object**.

Figure 22(b) is the contour of a leukocyte nucleus detected by the LNS method from the image in Figure 22(a). The ***R = 0.56 ***of the object ***obj ***indicated by a arrow in Figure 22(b) is less than ***r_A _***(in this example, ***r_A _***is set to ***0.7***), so ***obj ***needs splitting. Figure 22(c) shows the ***obj***. Until the fifth run in eroding ***obj***, ***obj ***is split into two sub-objects ***obj_1 _***and ***obj_2_***, displayed in Figure 22(d). Then, ***obj_1 _***is repeatedly split. After two eroding runs, ***obj_1 _***is separated into two objects ***obj_11 _***and ***obj_12_***, demonstrated in Figure 22(e). ***Obj_cut*() **continually severs ***obj_11_***. Figure 22(f) is the ***obj_11 _***after one eroding run. After two eroding runs, ***obj_11 _***will disappear. Hence, the algorithm runs dilation operation once on ***obj_11 _***in Figure 22(f); Figure 22(g) is the result of this run. Next, ***Obj_cut*() **tries to split ***obj_12_***. Since ***obj_12 _***will disappear after executing one erosion operation, no erosion and no dilation will be executed on ***obj_12_***. Afterward, ***Obj_cut*() **goes to erode ***obj_2_***. ***obj_2 _***vanishes after running erosion twice on ***obj_2_***. Figure 22(h) is the ***obj_2 _***after applying erosion operation once to ***obj_2_***. Therefore, ***Obj_cut*() **runs dilation operation once on ***obj_2_***; Figure 22(i) shows the final result of running ***Obj_cut*() **on original ***obj***.

After executing ***Obj_cut*(*obj*)**, some pixels in the original ***obj ***may not appear in the divided objects. Each of the unclassified pixels will be assigned to one of the divided objects. The LC method will compute the distance between every unclassified pixel ***p ***and each contour pixel in the divided objects and assigns ***p ***to the divided object, one of whose contour pixels is closest to ***p***. Afterwards, Figure 22(i) is converted into Figure 22(j). Figure 22(k) is the original image drawn on the divided object contours.

### Genetic-Based Parameter Detector (GBPD)

The performance of the LNS method is deeply affected by the values of ***r_s_***, ***r_G_***, ***r_t_***, ***r_r_***, ***t_1_***, ***t_2_***, ***t_3_***, ***t_4_***, ***t_5_***, and ***t_6_***. In this paper, a genetic-based parameter detector (GBPD) is employed to determine the most suitable values of ***r_s_***, ***r_G_***, ***r_t_***, ***r_r_***, ***t_1_***, ***t_2_***, ***t_3_***, ***t_4_***, ***t_5_***, and ***t_6_***.

A genetic algorithm (GA) [16] is a heuristic optimization method in which the set of possible solutions is considered a population of individuals. The adaptation degree of an individual to its environment is specified by its fitness. The coordinate of an individual in the search space is represented by a chromosome. A gene is a subsection of a chromosome that encodes the value of a single parameter being optimized. A genetic algorithm derives from evolutionary theory, so that, given a certain population, only the individuals adapting well to their environment can survive and transmit their characteristics to their descendants. Basically, a genetic algorithm consists of three major operations: selection, crossover, and mutation. Selection evaluates all individuals and only those most adaptable to their environment can survive. Crossover recombines the genetic material of two individuals to form new combinations with the potential for a better performance. Mutation induces changes in a small number of chromosomal units to maintain sufficient population diversity during the optimization process.

GBPD utilizes a binary string, concatenated by ten binary substrings ***s_s_***, ***s_G_***, ***s_t_***, ***s_r_***, ***s_1_***, ***s_2_***, ***s_3_***, ***s_4_***, ***s_5_***, and ***s_6_***, respectively comprised of $n_{r_{s}}$, $n_{r_{G}}$, $n_{r_{t}}$, $n_{r_{r}}$, ***n_1_***, ***n_2_***, ***n_3_***, ***n_4_***, ***n_5_***, and ***n_6 _***binary bits, to represent a chromosome ***Ch***. ***s_s_, s_G_, s_t_***, ***s_r_***, ***s_1_***, ***s_2_***, ***s_3_***, ***s_4_***, ***s_5_***, and ***s_6 _***are designated to describe the corresponding values of ***r_s_***, ***r_G_***, ***r_t_***, ***r_r_***, ***t_1_***, ***t_2_***, ***t_3_***, ***t_4_***, ***t_5_***, and ***t_6_***. For each chromosome ***Ch***, ***r_s_***, ***r_G_***, ***r_t_***, ***r_r_***, ***t_1_***, ***t_2_***, ***t_3_***, ***t_4_***, ***t_5_***, and ***t_6 _***are encoded as

$$\begin{array}{l} r_{s}=0.1+(n_{r_{s}}^{'}-1)\times 0.1,\\ r_{G}=1.5+(n_{r_{G}}^{'}-1)\times 0.1,\\ r_{t}=0.1+(n_{r_{t}}^{'}-1)\times 0.1,\\ r_{r}=0.1+(n_{r_{r}}^{'}-1)\times 0.1,\\ t_{1}=20+(n_{1}^{'}-1),\\ t_{2}=250+(n_{2}^{'}-1)\times 5,\\ t_{3}=20+(n_{3}^{'}-1),\;\text{and}\\ t_{4}=600+(n_{4}^{'}-1)\times 10,\\ t_{5}=n_{5}^{'}\times 0.05\\ t_{6}=n_{6}^{'}\times 0.05 \end{array}$$

where ${n^{'}}_{r_{s}}$, ${n^{'}}_{r_{G}}$, ${n^{'}}_{r_{t}}$, ${n^{'}}_{r_{r}}$, ${n^{'}}_{1}$, ${n^{'}}_{2}$, ${n^{'}}_{3}$, ${n^{'}}_{4}$, ${n^{'}}_{5}$, and ${n^{'}}_{6}$ are the number of ***1***-bits in ***s_s_, s_G_, s_t_***, ***s_r_***, ***s_1_***, ***s_2_***, ***s_3_***, ***s_4_***, ***s_5_***, and ***s_6_***, respectively.

GBPD applies the accumulated historic data to train the most appropriate ***r_s_***, ***r_G_***, ***r_t_***, ***r_r_***, ***t_1_***, ***t_2_***, ***t_3_***, ***t_4_***, ***t_5_***, and ***t_6 _***via a genetic algorithm. The manually drawn leukocyte nuclei contours are considered a collection of ground truths. GBPD uses the average relative foreground area error (RAE) as the measure of fitness of a chromosome based on the ***r_s_***, ***r_G_***, ***r_t_***, ***r_r_***, ***t_1_***, ***t_2_***, ***t_3_***, ***t_4_***, ***t_5_***, and ***t_6 _***encoded by the chromosome.

GBPD first randomly generates ***N ***chromosomes, each consisting of $n_{r_{s}}$, $n_{r_{G}}$, $n_{r_{t}}$, $n_{r_{r}}$, ***n_1_***, ***n_2_***, ***n_3_***, ***n_4_***, ***n_5_***, and ***n_6 _***binary bits. To evolve the best solution, the genetic algorithm repeatedly executes mutation, crossover, and selection operations until the relative fitness of the reserved chromosomes are similar.

In mutation operation, for each of the ***N ***reserved chromosomes, GBPD uses a random number generator to specify one bit ***b ***for each of ***s_s_, s_G_, s_t_***, ***s_r_***, ***s_1_***, ***s_2_***, ***s_3_***, ***s_4_***, ***s_5_***, and ***s_6_***. ***b ***is then replaced by ***¬b ***to generate a new chromosome, where ***¬ ***signifies the operator "**NOT**."

In crossover operation, similarly a random number generator is used to designate ***N' ***pairs of chromosomes from the ***N ***reserved chromosomes. Let ***Ch*[*i..j*] **be the substring consisting of the ***i***^th ^to ***j***^th ^bits in ***Ch, S *= {*0*, **$n_{r_{s}}$, $n_{r_{G}}$, $n_{r_{t}}$, $n_{r_{r}}$**, *n_1_*, *n_2_*, *n_3_*, *n_4_*, *n_5_*, *n_6_*} **be an ordered set, and ***e_i _***be the ***i***^th ^element in ***S***. For each chromosome pair (***Ch_1_***, ***Ch_2_*)**, the genetic algorithm concatenates

$$\overset{\mathrm{10}}{\underset{1}{⊗}}(Ch_{1}\left[\left(1+\sum_{j=0}^{i-1}e_{j}\right)..\left(\sum_{j=0}^{i-1}e_{j}+\left\lfloor \frac{e_{i}}{2}\right\rfloor \right)\right]⊗Ch_{2}\left[\left(\sum_{j=0}^{i-1}e_{j}+\left\lfloor \frac{e_{i}}{2}\right\rfloor +1\right)..\sum_{j=0}^{i}e_{j}\right])$$

into a new chromosome, and concatenates

$$\overset{\mathrm{10}}{\underset{1}{⊗}}(Ch_{2}\left[\left(1+\sum_{j=0}^{i-1}e_{j}\right)..\left(\sum_{j=0}^{i-1}e_{j}+\left\lfloor \frac{e_{i}}{2}\right\rfloor \right)\right]⊗Ch_{1}\left[\left(\sum_{j=0}^{i-1}e_{j}+\left\lfloor \frac{e_{i}}{2}\right\rfloor +1\right)..\sum_{j=0}^{i}e_{j}\right])$$

into another new chromosome, where **⊗ **represents a concatenation operator.

In selection operation, ***N ***optimal chromosomes are selected from the ***N ***chromosomes reserved in the previous iteration and ***N ***as well as ***2*×*N' ***chromosomes created in the mutation and crossover operations according to their fitness. Three major operations (mutation, crossover, and selection) need to be continuously performed until the related fitness of the reserved ***N ***chromosomes is very close or the number of iterations is equal to the specified maximum number of generations.

**Figure 23:**
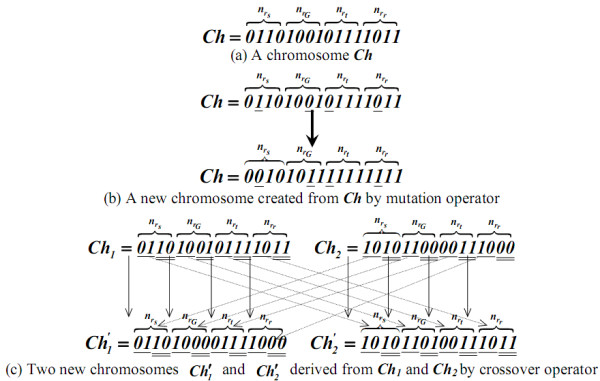
**An example for GBPD**.

Figure 23(a) shows a chromosome ***Ch ***with $n_{r_{s}}$**= *4***, $n_{r_{G}}$**= *4***, $n_{r_{t}}$**= *4***, and $n_{r_{r}}$ = ***4***; derived from ***Ch***, ***r_s _= 0.2***, ***r_G _= 1.6***, ***r_t _= 0.3 ***and ***r_r _= 0.3***. For convenience to describe, in this example, we assure that ***Ch ***only consists of four substrings ***s_s_, s_G_, s_t_***, and ***s_r_***. Figure 23(b) demonstrates a new chromosome created from ***Ch ***by a mutation operator, where the bits underlined are the randomly selected bits ***b***'s. Two new chromosomes $C{h^{'}}_{1}$ and $C{h^{'}}_{2}$ generated from the two chromosomes ***Ch1 ***and ***Ch2 ***through the crossover operator as shown in Figure 23(c).

## Authors' contributions

DCH and YKC conceived the study. DCH designed the approach and performed the computational analysis with YKC, ZHZ, and KDH. DCH and YKC supervised the work and tested the program. DCH, YKC, and MHT wrote the manuscript. MHT prepared the samples and collected the data together with ZHZ and KDH. MHT contributed analyzing experimental studies. All authors read and approved the final manuscript. YKC and MHT contributed equally and are the first authors as well as listed in alphabetical order.

## References

[B1] Timby BK Smith NE Introductory Medical-Surgical Nursing 2006 Nine Lippincott Williams & Wilkins

[B2] Human Physiology and Anatomy Blood Cell Histology http://www.unomaha.edu/hpa/blood.html

[B3] Bagby GC Goldman L, Ausiello D Leukopenia and Leukocytosis Cecil Medicine 2007 23 Philadelphia, Pa: Saunders Elsevier

[B4] Scientific Psychic The Hematologist http://www.scientificpsychic.com/mind/whitecells.html

[B5] Bailey SC Head JF Greengard O "Neutrophil Maturation and Hypersegmentation Promoted in Normal Bone Marrow by a Carcinoma-Elaborated Protein Factor," American Journal of Hematology 2006 31 3 159 165 10.1002/ajh.2830310304 2662758

[B6] Baxes GA Digital Image Processing: Principles and Applications 1994 New York: John Wiley & Sons

[B7] Theera-Umpon N Dhompongsa S "Morphological Granulometric Features of Nucleus in Automatic Bone Marrow White Blood Cell Classification," IEEE Transactions on Information Technology in Biomedicine 2007 11 3 353 359 10.1109/TITB.2007.892694 17521086

[B8] Pingkum Y Zhou X Shah M Wong STC "Automatic Segmentation of High-Throughput RNAi Fluorescent Cellular Images," IEEE Transactions on Information Technology in Biomedicine 2008 12 1 109 117 10.1109/TITB.2007.898006 18270043 PMC2846541

[B9] Tang C Ewert B "Automatic Tracking of Neural Stem Cells," Proceedings of WDIC2005, Brisbane, Australia 2005 61 66

[B10] Hamghalam M Motameni M Kelishomi AE "Leukocyte Segmentation in Giemsa-stained Image of Peripheral Blood Smears Based on Active Contour," International Conference on Signal Processing Systems 2009 103 106 full_text

[B11] Liu J Leong TY Chee KB Tan BP Shuter B Wang SC "Set-Based Cascading Approaches for Magnetic Resonance (MR) Image Segmentation (SCAMIS)," AMIA Annual Symposium proceedings 2006 504 508 17238392 PMC1839603

[B12] Gonzalez RF Wintz P Digital image processing 1992 3 Addison-Wesley

[B13] Yang-Mao SF Chan YK Chu YP "Edge Enhancement Nucleus and Cytoplast Contour Detector of Cervical Smear Images," IEEE Transactions on Systems, Man, and Cybernetics-PART B: Cybernetics 2008 38 2 353 366 10.1109/TSMCB.2007.912940 18348920

[B14] Gonzalez R Woods R Digital image processing 2002 Englewood Cliffs, NJ: Prentice-Hall

[B15] Otsu N "A Threshold Selection Method from Gray Level Histogram," IEEE Transactions on Systems, Man, and Cybernetics - B 1978 8 1 62 66 10.1109/TSMC.1978.4309832

[B16] Man KF Tang KS Kwong S Genetic Algorithms: Concepts and Designs 1999 Springer-Verlag, New York

